# Supplementary material for: Construction of Viable Soil Defined Media Using Quantitative Metabolomics Analysis of Soil Metabolites
Source: Front Microbiol. 2017 Dec 22;8:2618. doi: 10.3389/fmicb.2017.02618 (PMC5744445; doi:10.3389/fmicb.2017.02618)
Supplement: Supplementary file 4 [file Image1.PDF]

Supplementary Figures 1-2

Construction of viable soil defined media using quantitative metabolomics analysis of soil metabolites

Stefan Jenkins, Tami L. Swenson, Rebecca Lau, Andrea M. Rocha, Alex Aaring, Terry C. Hazen, Romy Chakraborty, Trent Northen

*Corresponding author: trnorthen@lbl.gov

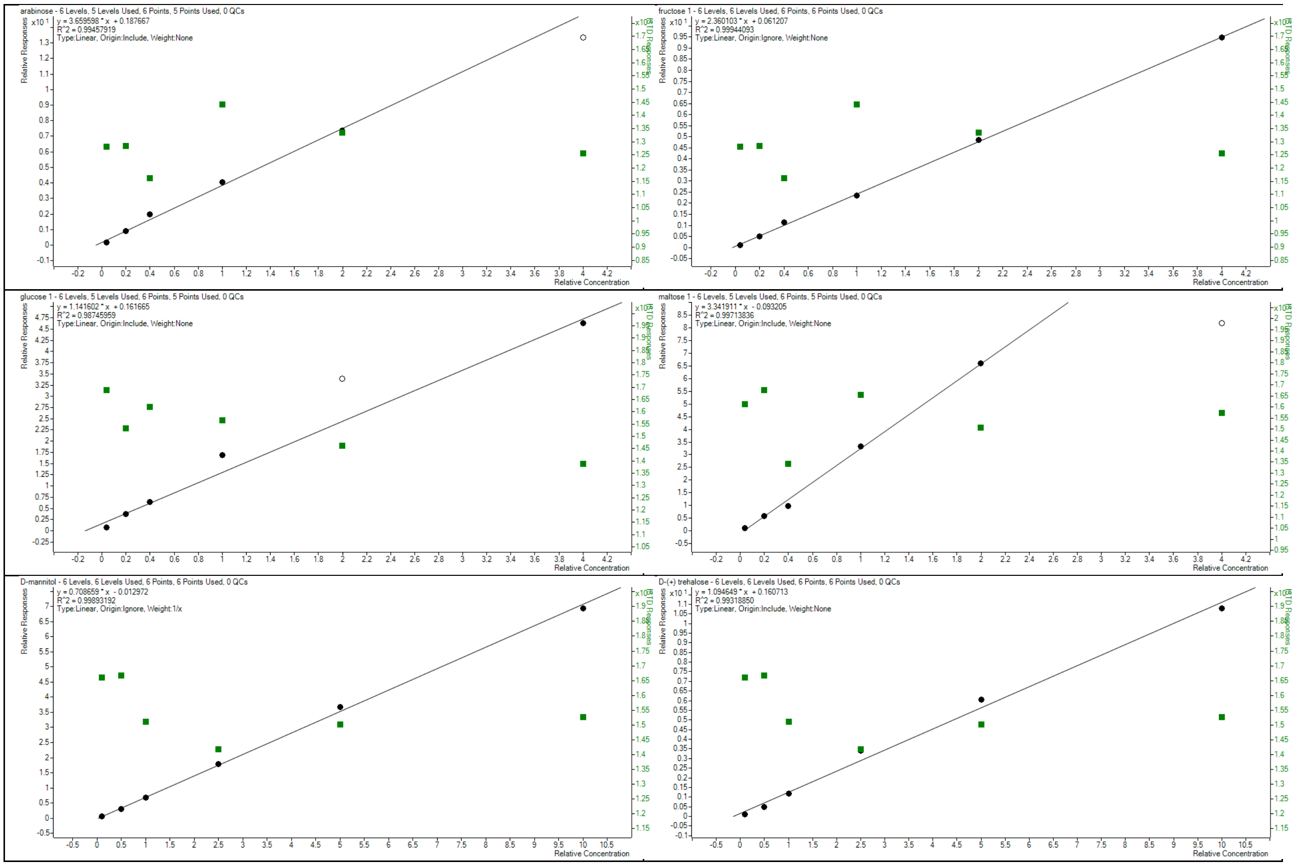

Supp. Figure 1A. GC/MS calibration curves for sugars quantified in soil organic matter extracts (green squares- internal standard, black circle- calibration sample).

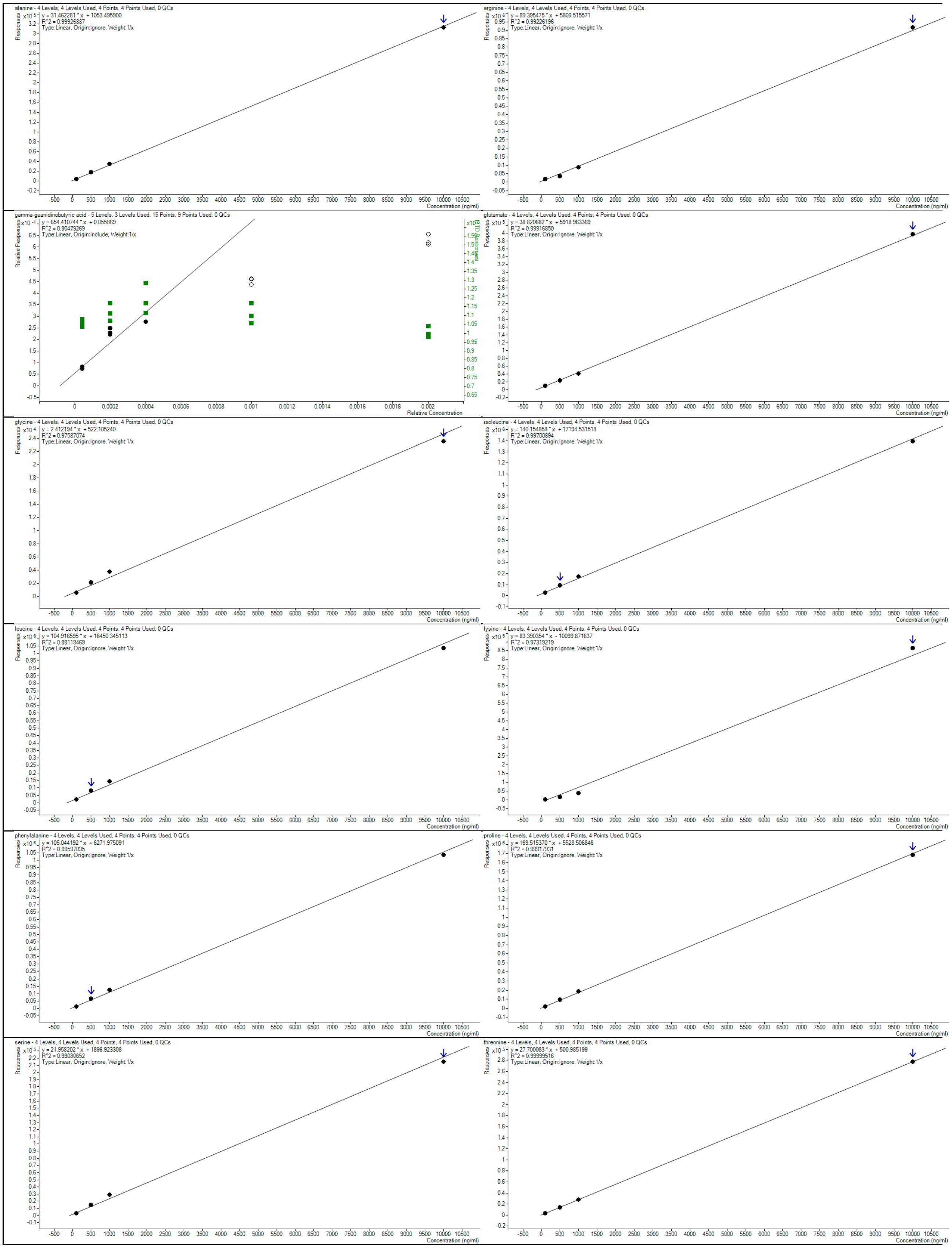
alanine - 4 Levels, 4 Levels Used, 4 Points, 4 Points Used, 0 QCs
y = 31.462281 * x + 1053.495900
R^2 = 0.99926887
Type:Linear, Origin:Ignore, Weight:1/x
Responses
Concentration (ng/ml)
arginine - 4 Levels, 4 Levels Used, 4 Points, 4 Points Used, 0 QCs
y = 89.395475 * x + 5809.515571
R^2 = 0.99226196
Type:Linear, Origin:Ignore, Weight:1/x
Responses
Concentration (ng/ml)
gamma-guanidinobutyric acid - 5 Levels, 3 Levels Used, 15 Points, 9 Points Used, 0 QCs
y = 654.410744 * x + 0.055869
R^2 = 0.90479269
Type:Linear, Origin:Include, Weight:1/x
Relative Responses
ISTD Responses
Relative Concentration
glutamate - 4 Levels, 4 Levels Used, 4 Points, 4 Points Used, 0 QCs
y = 38.820682 * x + 5918.963369
R^2 = 0.99916850
Type:Linear, Origin:Ignore, Weight:1/x
Responses
Concentration (ng/ml)
glycine - 4 Levels, 4 Levels Used, 4 Points, 4 Points Used, 0 QCs
y = 2.412194 * x + 522.185240
R^2 = 0.97587074
Type:Linear, Origin:Ignore, Weight:1/x
Responses
Concentration (ng/ml)
isoleucine - 4 Levels, 4 Levels Used, 4 Points, 4 Points Used, 0 QCs
y = 140.154858 * x + 17194.531518
R^2 = 0.99700894
Type:Linear, Origin:Ignore, Weight:1/x
Responses
Concentration (ng/ml)
leucine - 4 Levels, 4 Levels Used, 4 Points, 4 Points Used, 0 QCs
y = 104.916595 * x + 16450.345113
R^2 = 0.99119469
Type:Linear, Origin:Ignore, Weight:1/x
Responses
Concentration (ng/ml)
lysine - 4 Levels, 4 Levels Used, 4 Points, 4 Points Used, 0 QCs
y = 83.390354 * x - 10099.871637
R^2 = 0.97319219
Type:Linear, Origin:Ignore, Weight:1/x
Responses
Concentration (ng/ml)
phenylalanine - 4 Levels, 4 Levels Used, 4 Points, 4 Points Used, 0 QCs
y = 105.044192 * x + 6271.975091
R^2 = 0.99597835
Type:Linear, Origin:Ignore, Weight:1/x
Responses
Concentration (ng/ml)
proline - 4 Levels, 4 Levels Used, 4 Points, 4 Points Used, 0 QCs
y = 169.515370 * x + 5528.506846
R^2 = 0.99917931
Type:Linear, Origin:Ignore, Weight:1/x
Responses
Concentration (ng/ml)
serine - 4 Levels, 4 Levels Used, 4 Points, 4 Points Used, 0 QCs
y = 21.958202 * x + 1896.923308
R^2 = 0.99080652
Type:Linear, Origin:Ignore, Weight:1/x
Responses
Concentration (ng/ml)
threonine - 4 Levels, 4 Levels Used, 4 Points, 4 Points Used, 0 QCs
y = 27.700083 * x + 500.985199
R^2 = 0.99999516
Type:Linear, Origin:Ignore, Weight:1/x
Responses
Concentration (ng/ml)

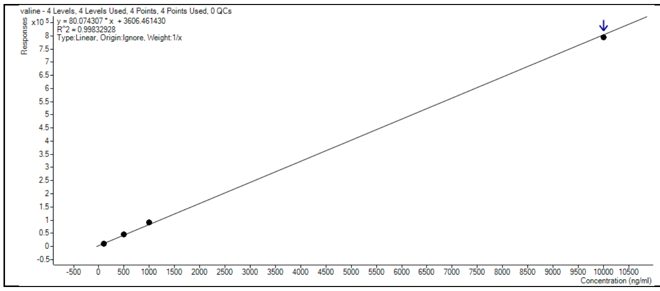

Supp. Figure 1B. LC/QQQ-MS calibration curves for amino acids and amino acid derivative quantified in soil organic matter extracts.

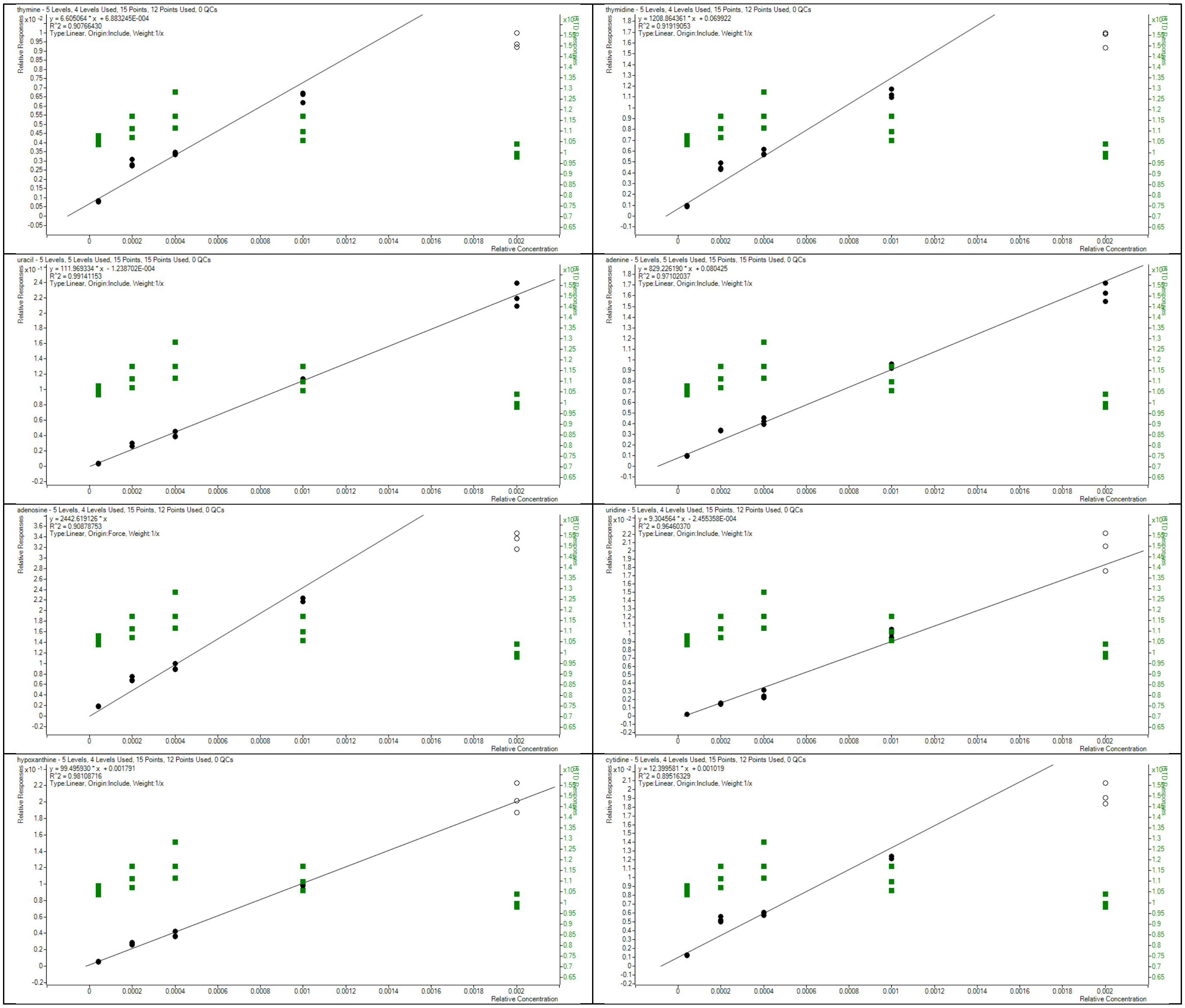

Supp. Figure 1C. LC/QQQ-MS calibration curves for nucleobases and nucleosides quantified in soil organic matter extracts (green squares- internal standard, black circle- calibration sample).

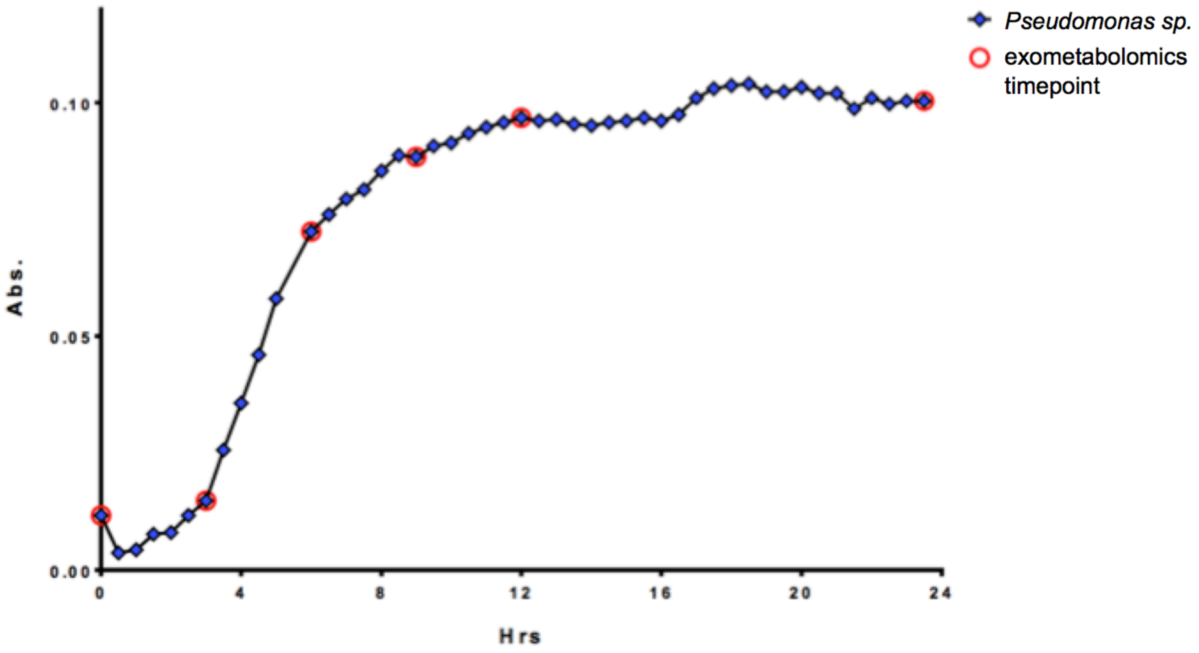

Supp. Fig. 2. 24 h growth curve of *Pseudomonas sp*. FW300-N2E2 cultivated in SDM1-10x medium. Exometabolomic sampling time points were taken at 0, 3, 6, 9, 12 and 24 h as indicated by red circles. Absorbance indicates OD_{600} after media control blank subtraction.
